# Ultrasound combined with SDF‐1α chemotactic microbubbles promotes stem cell homing in an osteoarthritis model

**DOI:** 10.1111/jcmm.15706

**Published:** 2020-08-17

**Authors:** Xi Xiang, Hui Liu, Liyun Wang, Bihui Zhu, Lang Ma, Fangxue Du, Ling Li, Li Qiu

**Affiliations:** ^1^ Department of Medical Ultrasound Laboratory of Ultrasound Imaging Drug West China Hospital of Sichuan University Chengdu China; ^2^ Department of Ultrasound The Affiliated Hospital of Southwest Medical University Luzhou China

**Keywords:** homing, mesenchymal stem cell, microbubble, osteoarthritis, stromal cell‐derived factor‐1, ultrasound

## Abstract

Osteoarthritis (OA) is a common joint disease in the middle and old age group with obvious cartilage damage, and the regeneration of cartilage is the key to alleviating or treating OA. In stem cell therapy, bone marrow stem cell (BMSC) has been confirmed to have cartilage regeneration ability. However, the role of stem cells in promoting articular cartilage regeneration is severely limited by their low homing rate. Stromal cell‐derived factor‐1α (SDF‐1α) plays a vital role in MSC migration and involves activation, mobilization, homing and retention. So, we aim to develop SDF‐1α‐loaded microbubbles MB(SDF‐1α), and to verify the migration of BMSCs with the effect of ultrasound combined with MB(SDF‐1α) in vitro and in vivo. The characteristics of microbubbles and the content of SDF‐1α were examined in vitro. To evaluate the effect of ultrasound combined with chemotactic microbubbles on stem cell migration, BMSCs were injected locally and intravenously into the knee joint of the OA model, and the markers of BMSCs in the cartilage were detected. We successfully prepared MB(SDF‐1α) through covalent bonding with impressive SDF‐1α loading efficacy loading content. In vitro study, ultrasound combined with MB(SDF‐1α) group can promote more stem cell migration with highest migrating cell counts, good cell viability and highest CXCR4 expression. In vivo experiment, more BMSCs surface markers presented in the ultrasound combined with MB(SDF‐1α) group with or without exogenous BMSCs administration. Hence, ultrasound combined with MB(SDF‐1α) could promote the homing of BMSCs to cartilage and provide a novel promising therapeutic approach for OA.

## 1 INTRODUCTION

Osteoarthritis (OA) is a group of common joint diseases in the elderly, characterized by articular cartilage destruction, excessive joint stiffness, pain, and spasm. OA often involves multiple joints, mostly in the knee joint, elbow joint, hip joint, hand joint. According to the statistical analysis of the United Nations, by 2050, 130 million people worldwide are expected to develop the disease, and 40 million of them will develop into severe OA.^1^ There is tremendous economic pressure in the treatment and management of OA, and the quality of life of patients can be significantly reduced.

The lesions of OA are mainly concentrated on articular cartilage. Articular cartilage is a transparent lining on the joint surface of the bone that cushions external impact and effectively reduces friction between the bones for smooth, painless joint movement.^2^ The key to treating OA is to repair the damaged articular cartilage. However, treatment for articular cartilage defects still presents a major therapeutic challenge because of its hypocellular nature, vulnerability to injury and poor capacity to spontaneous regeneration.

Generally speaking, the existing treatment methods include lifestyle change, painkilling drugs, intra‐articular injection of hormones, hyaluronic acid and other symptomatic treatments, which cannot prevent the progression of early and mid‐stage lesions. In the late stage of the disease, joint replacement surgery can achieve better results, but it may still cause serious complications, such as postoperative infection, loosening of the prosthesis, and fractures around the prosthesis.

Nowadays, tissue engineering techniques have demonstrated the possibility of using mesenchymal stem cells (MSCs) for the regeneration of a variety of tissues. Bone marrow mesenchymal stem cells (BMSCs) can differentiate into tissues such as bone and cartilage, and have great potential in cartilage regeneration.^3^ The researchers used MSCs in the treatment of OA, with or without drugs or stents, and achieved a certain degree of efficacy.^4^, ^5^, ^6^, ^7^, ^8^, ^9^, ^10^ All of the above studies have focused on the application of exogenous MSCs to local joints, but OA is a disease involving multiple joints, and it would be better if it could be treated systemically. In the current researches on systemic application of MSCs, the low homing rate of MSCs in target organs significantly affects the repair of target organs. Effective MSC therapy requires that MSCs reach the injured site.^11^ So, increasing the number of stem cells in target organs, and promoting the homing to target organs is the key to promote tissue regeneration.^12^, ^13^

Stromal cell‐derived factor‐1 (SDF‐1) is an extremely important chemokine for MSCs. It includes two subtypes, SDF‐1α and SDF‐1β, and SDF‐1α plays a major role in chemotaxis. SDF‐1 is well known for MSCs activation, mobilization, homing and also retention.^14^ CXCR4 is a chemokine receptor that presents on the surface of stem cells. SDF‐1α and CXCR4 form the most important biological axis to promote homing of stem cells.^15^, ^16^, ^17^ Increasing the expression of SDF‐1α or CXCR4 can raise the homing efficiency of MSCs.^18^, ^19^

Microbubble (MB) is a type of monolayer bubble having a diameter between 0.5 and 10 μm. It is often used as a contrast agent and a targeted drug in medical imaging,^20^ such as antibodies, polypeptides or transferrin vectors^21^, ^22^, ^23^ and gene delivery vectors.^20^ Ultrasound‐targeted microbubble destruction (UTMD) has been confirmed to increase the homing of transplanted MSCs to target organs,^24^ and also provide a suitable environment for the survival of migrated stem cells.^25^ In addition, UTMD can also promote the involvement of endocrine factors in stem cell homing.^24^

Herein, in this study, we designed a chemotactic microbubble MB(SDF‐1α) through the covalent bonding of SDF‐1α to the microbubble shell, based on the perspective of SDF‐1/CXCR4 axis regulation of stem cell homing. The chemotactic microbubbles are locally released SDF‐1α to the target tissue under the irradiation of ultrasound. Next, BMSCs were injected intravenously, and pathological detection of BMSCs surface markers was performed for further detection and analysis. In this way, we aimed to potentiate the BMSCs homing to the cartilage in order to provide a promising approach for effective stem cell therapy for OA.

## 2 MATERIALS AND METHODS

### 2.1 Main Materials

Disaturated phosphatidyl choline (DSPC), distearoyl phosphatidyl choline‐ polyethylene glycol 2000‐ carboxyl (DSPE‐PEG2000‐COOH) and polyethylene glycol 4000 (PEG‐4000) were purchased from AVT. Palmic acid (PA) was purchased from Aladdin Bio‐Chem Technology. Recombinant rat SDF‐1α was purchased from GenScript Biotech Corporation. N‐hydroxysulphosuccinimide sodium salt (sulfo‐NHS), 1‐Ethyl‐3‐(3‐dimethylaminopropyl) carbodiimide (EDC), fluorescein isothiocyanate (FITC), phosphate buffer saline (PBS), AMD3100, and CCK‐8 kit were purchased from Sigma‐Aldrich. Rat bone marrow stem cells (BMSC) were purchased from Cyagen Biotechnology Co., Ltd. Transwell chamber and six‐well plate were purchased from Corning. APC‐CXCR4 antibody was purchased from Thermo Fisher. Anti‐rat‐CXCR4, anti‐rat‐CD73 and anti‐rat‐CD90/Thy 1 were purchased from Abcam. Perfluoropropane (SF_6_) was purchased from Jietong Gas Technology Co., Ltd.

Adult male Sprague‐Dawley (SD) rats (weighed 200‐250 g) were supplied by Chengdu Dashuo Experimental Animal Co., Ltd. In this experiment, all animal procedures were carried out in accordance with the protocol of the Animal Research Committee of Sichuan University.

### 2.2 Preparation of MB and MB(SDF‐1α)

The common MBs that not containing SDF‐1α were basically prepared according to the methods of other researchers,^26^ and some improvements of the protocol were also made. First, the composite membrane of DSPC and PA was prepared. Then mix the materials including DSPC + PA, DSPE‐PEG2000‐COOH, and PEG‐4000 (mass ratio of 1:1:50) with ultrapure water. A 60°C water bath could help dissolve and mix thoroughly. The equal amount of the mixed solution is then dispensed into a vial and quickly lyophilized into a powder. The powder was then dissolved by the solution that consists of 50% glucose, propylene glycol, and glycerine with volume ratio of 8:1:1. After sealing the above‐mixed lipid solution, the air in the bottle is removed by a gas suction device, and then the SF_6_ is rapidly filled in. Finally, place the vial in the shaker for oscillation for 45 seconds at a frequency of 4300/min and amplitude of 15 mm. According to this method, common MBs are obtained.

**FIGURE 1:**
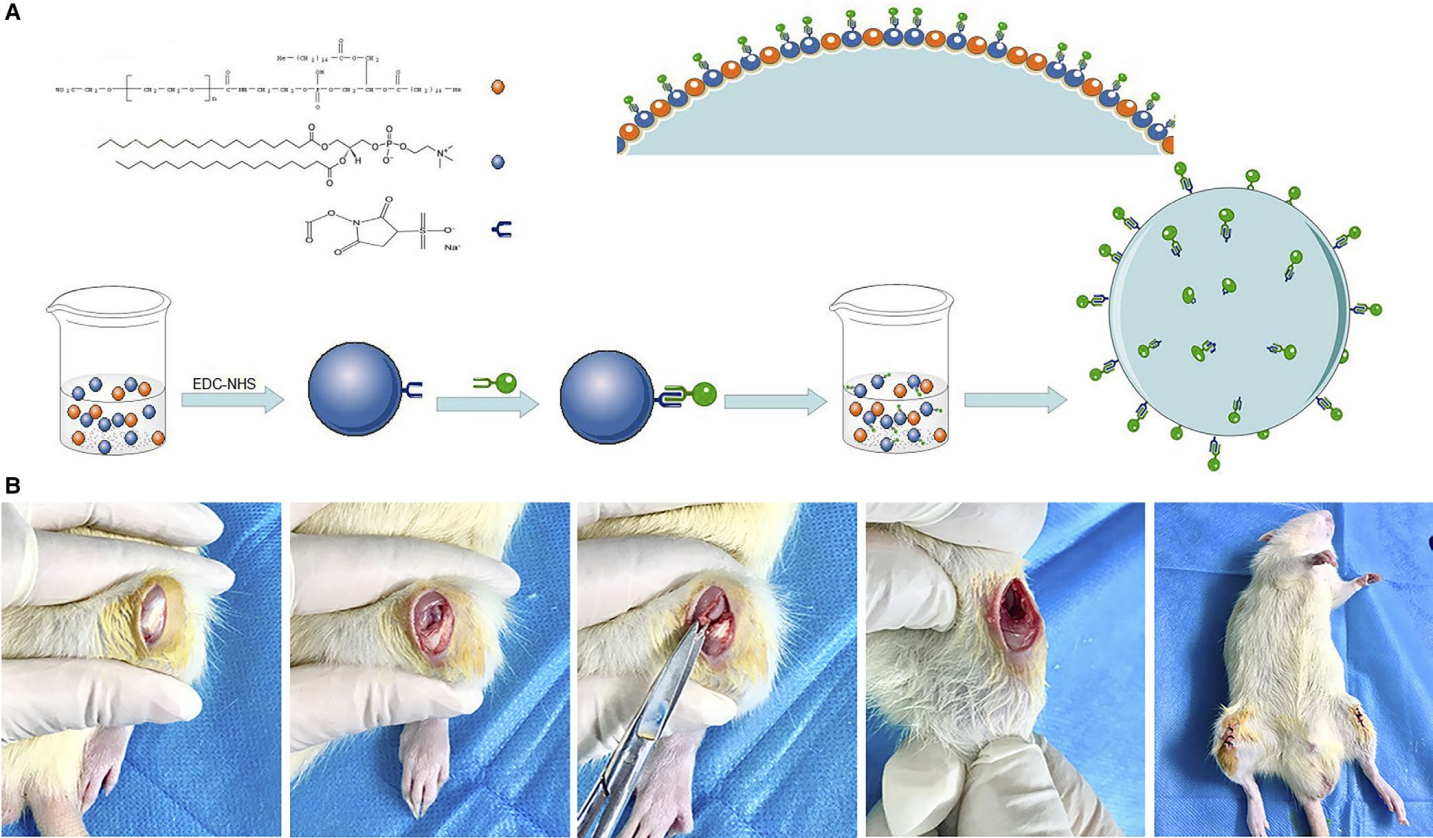
Preparation of MB(SDF‐1α) and OA animal model. A, Flow chart of chemotaxis microbubble MB(SDF‐1α) preparation. The carboxyl group carried by the lipid molecule and the amino group of SDF‐1 are linked by a covalent bond. After all, chemotactic microbubbles containing C3F8 and SDF‐1α linked in the outer shell were prepared. B, The modified Hulth's modelling method for OA. During the operation, the medial meniscus is removed, and the anterior cruciate ligament is severed, causing joint instability and eventually osteoarthritis. An OA model was established on bilateral knees

MB(SDF‐1α) is synthesized by a covalent bond through the carboxyl group of the lipid shell and the amino group carried by SDF‐1α. Briefly, the carboxyl group of DSPE‐PEG2000‐COOH needed to be activated by EDC/NHS. That is, EDC (5 μL, 50 mg/mL) and NHS (5 μL, 100 mg/mL) were respectively added to the MB solution before gas replacement. After 15 minutes of activation, 20 μL SDF‐1α solution containing 20 μg SDF‐1α were added and another 2 hours were needed for the reaction. The resultant solution was treated following the same protocol as that used for common MBs to form the preliminary MB(SDF‐1α) (Figure 1A). Finally, the preliminary MB(SDF‐1α) needed to be purified by centrifugation at 1000 rpm for 5 minutes to remove the free SDF‐1α which was not loaded to the MBs, as well as the coupling agents. To evaluate the efficiency of conjunction between SDF‐1α and MBs, we labelled SDF‐1α with FITC prior to the experiment.

### 2.3 Physical properties and characterization of MB(SDF‐1α) and MB

The concentration and particle size of MB(SDF‐1α) and MB were determined with Beckman Coulter (Multisizer 4e) at hours 2, 8 and 24, respectively. The morphology, size, and distribution of the two microbubbles were observed using an optical microscope (AX10 imager A2/AXP10 cam HRC; Zeiss). FITC fluorescence expression was observed using an inverted fluorescence microscope (OBSERVER D1/AX10 cam HRC; Zeiss) and flow cytometry to determine the conjunction rate of SDF‐1α, respectively. In addition, enzyme‐linked immune sorbent assay (ELISA) (R&D Systems, Inc) was applied to quantitatively determine the loaded SDF‐1α. Finally, the SDF‐1α encapsulation efficiency and loading content were calculated, respectively.

### 2.4 Preparation of Bone marrow mesenchymal stem cells

Rat BMSCs were purchased from Cyagen Biotechnology Co., Ltd. These cells have been identified as bone marrow MSCs by flow cytometry and adipogenic and osteogenic differentiation induction before the sale. All BMSCs were passed to the third generation and prepared for use. In subsequent experiments, the concentration of stem cells was adjusted according to the needs of the experiment.

### 2.5 BMSCs migration experiment in vitro

This part of experiment included two sections, chemotaxis experiment, and chemotaxis inhibition experiment. In the chemotaxis experiment, adjust the BMSCs concentration to 2 × 10^5^/mL using the basal medium. All of the experiments were carried out in six‐well plates. The wells were divided into upper chambers and lower chambers. Add 1 mL BMSCs to all upper chambers respectively. And different experimental operations were performed on the lower chambers according to different groups. There are five groups in this study, control group, SDF‐1α group, MB group, MB(SDF‐1α) group and MB(SDF‐1α) + ultrasound (US) group (Table 1). The ultrasonic conditions of group five are duty cycle 20%, sound intensity 1 W/cm^2^, irradiation time 30 seconds, and frequency 1 MHz. After the operation of each group was completed, they were incubated at 37°C, 5% CO_2_ for 8 hours. Then, the cells that migrated to the lower side of the transwell were stained with 0.1% crystal violet for 20 minutes and then washed with water. Finally, the cells were tested as follows: cell counts, cell activity assay using CCK‐8 kit, flow cytometry to detect the existence of CXCR4 and qRT‐PCR to determine the CXCR4 expression.

**TABLE 1:** Grouping in in vitro experiments

Groups	Upper chamber	Lower chamber
(1) Control	1 mL BMSCs	Stem cell complete medium 1.5 mL
(2) SDF‐1α group	1 mL BMSCs	150 ng SDF‐1α + complete medium 1.5 mL
(3) MB group	1 mL BMSCs	Add MB to the same volume of MB solution as MB(SDF‐1α) to 1.5 mL
(4) MB(SDF‐1α) group	1 mL BMSCs	MB(SDF‐1α) solution containing 150 ng of SDF‐1α, supplemented with medium to 1.5 mL
(5) MB(SDF‐1α) + US group	1 mL BMSCs	MB(SDF‐1α) solution containing 150 ng of SDF‐1α, supplemented with medium to 1.5 mL

In the chemotaxis inhibition experiment, the key point was to incubate BMSCs with the CXCR4 receptor antagonist AMD3100 (5 μg/mL) for 30 minutes to block CXCR4. Then the group experiments were performed after adjusting the cell concentration. And finally, the numbers of migrated cells in each group were counted.

### 2.6 Animal preparation

A total of 144 adult male SD rats (weighed 200‐250 g) were selected to establish bilateral knee OA models by modified Hulth's modelling method (Figure 1B). Briefly, rats were anesthetized by intraperitoneal injection of 0.3% sodium pentobarbital solution at a dose of 1 mL/100 g. The rats were placed supine on the operating table, and the area in front of the knee was prepared and disinfected using iodophor. Subsequently, the knee joint of the rat was kept at the maximum knee flexion, and the skin and the subcutaneous layer were longitudinally cut to fully expose the patellar tendon. Open the junction of the tendon and the muscle in the outer side of the patellar tendon, and cut it deeply into the joint cavity. The knee joint is then laid flat, then longitudinally cut along the medial side of the knee, and the patellar tendon was turned to the outside of the joint to expose the femoral trochlear. The medial meniscus of the knee joint was removed, and the anterior cruciate ligament was cut, while the posterior cruciate ligament was still preserved. A drawer test was performed on the knee joint of the rat to verify the stability of the joint. If the drawer test was positive, the operation was considered successful. All rats underwent bilateral knee arthroplasty and were housed in SPF animal laboratories. All experiments were performed according to the rules of the Animal Research Committee of Sichuan University.

### 2.7 Grouping of in vivo experiments and experimental procedures

The in vivo study was divided into two main parts: (a) verification of exogenous stem cell homing (Exogenous BMSCs group); (b) verification of possible endogenous stem cell homing (Non‐Exogenous BMSCs group). Briefly, the greatest difference between the two parts was the injection of exogenous BMSCs or not. When verifying exogenous BMSCs homing, the experiment was grouped as follows: (a) Control; (b) BMSCs; (c) BMSCs + US; (d) BMSCs + MB+US; (e) BMSCs + MB(SDF‐1α) + US; (f) BMSCs + MB(SDF‐1α) (IA, Intra‐articular injection) + US. All groups of BMSCs were injected from the tail vein, and the number of BMSCs injected per rat was 1 × 10^6^. While, when verifying the possible endogenous BMSCs homing, the experiment was grouped as follows: (a) Control; (b) MB; (c) MB + US; (d) MB(SDF‐1α); (e) MB(SDF‐1α) + US; (f) MB(SDF‐1α)(IA)+US. The detailed experiment procedures of different groups were shown in Table 2. After the OA models were established, rats were randomly assigned to each experimental group, 12 rats in each group. Except for the intra‐articular injection group, both MB and MB(SDF‐1α) were injected from the tail vein, and each rat was injected with 0.2 mL. Both microbubbles were stored after preparation in short time before the experiment. The in vivo experimental ultrasonic irradiation conditions were: duty ratio 30%, sound intensity 2 W/cm^2^, duration time 3 minutes. Rats were randomly sacrificed in batches on days 1, 2, and 7 after receiving different treatments. Finally, reserve the bilateral knee joints for subsequent tests.

**TABLE 2:** Grouping in vivo experiments

Sections	Groups	Experiment procedure
A. Exogenous BMSCs	(1) Control	None
	(2) BMSCs	Injection of BMSCs from the tail vein
	(3) BMSCs + US	Ultrasound irradiation at the knee joint after injecting BMSCs into the tail vein
	(4) BMSCs + MB+US	Ultrasound irradiation at the knee joint after injecting BMSCs and MB into the tail vein
	(5) BMSCs + MB(SDF‐1α) +US	Ultrasound irradiation at the knee joint after injecting BMSCs and MB(SDF‐1α) into the tail vein
	(6) BMSCs + MB(SDF‐1α)(IA)+US	After injecting BMSCs into the tail vein, MB(SDF‐1α) was injected into the knee joint, followed by ultrasound irradiation
B. Non‐Exoogenous BMSCs	(1) Control	None
	(2) MB	Injection of MB from the tail vein
	(3) MB + US	Ultrasound irradiation at the knee joint after injecting MB into the tail vein
	(4) MB(SDF‐1α)	Injection of MB(SDF‐1α) from the tail vein
	(5) MB(SDF‐1α) + US	Ultrasound irradiation at the knee joint after injecting MB(SDF‐1α) into the tail vein
	(6) MB(SDF‐1α)(IA)+US	Ultrasound irradiation after injecting MB(SDF‐1α) into the knee joint

Abbreviation: IA, Intra‐articular injection.

### 2.8 Pathological examination

The knee joint is inherently hard and cannot be sliced directly. It must be decalcified and then paraffin‐embedded. When the prepared wax block is sliced, one slice is collected at intervals of five pieces, and the slice thickness is 4 μm. Each group of knee joints received haematoxylin and eosin staining (HE staining), Safranin O‐Fast green staining and immunohistochemistry (IHC).

#### 2.8.1 HE staining

Haematoxylin and eosin staining is based on different eosinophilic and basophilic dyes of haematoxylin and eosin. Haematoxylin can dye acidic components such as nucleic acids into blue, while eosin can dye alkaline components such as proteins into red.^27^ HE staining can not only show the general morphology of articular cartilage in the OA model but also observe the cartilage defects in the joints and also assess the extent of inflammatory cell infiltration in OA.

#### 2.8.2 Safranin O‐Fast green staining

Safranin O is a basic dye that can be red in combination with acidic proteoglycans; fast green is an acid dye that combines with acidic collagen to appear green. The semi‐quantitative analysis of collagen components in the cartilage matrix can be performed by the image of Safranin O‐solid green staining, and then the modified Mankin's scoring standard is used to obtain the degeneration of cartilage tissue after comprehensive scoring.^28^ It can visually reflect the structure of articular cartilage, subchondral bone, and bone tissue, and is superior to HE staining in display structure.

#### 2.8.3 Immunohistochemistry

Immunohistochemistry was performed to compare stem cell marker expression between different groups of rats. CD73 and CD90 are markers for expression on the surface of MSCs. The CXCR4 existing on the surface of BMSCs is usually low in content, and it is a very important factor that affects the chemotaxis of the SDF‐1/CXCR4 axis. Therefore, we will also test the expression of CXCR4. The major antibodies were Anti‐CXCR4 antibody (ab124824; Abcam), Anti‐CD73 antibody (ab175396; Abcam), Anti‐CD90/Thy1 antibody [EPR3132] (ab92574; Abcam). After incubation with primary antibody and correspondent secondary antibody, all sections were collected and 10 fields were taken from each section under a fluorescence microscope (AX10 imager A2/AX10 cam HRC; Zeiss) and a computer image analysis system (Image‐Pro Plus; Media Cybernetics) was used for scores. Then data were expressed in terms of mean density and averaged after three scores.

### 2.9 Quantitative real‐time reverse transcription‐polymerase chain reaction

Quantitative real‐time reverse transcription‐polymerase chain reaction (qRT‐PCR) was used to analyse the expression of the chemokine receptor CXCR4 and the surface positive marker of BMSCs (CD73, CD90) in different groups. Briefly, RNA was first extracts and then transcribed into cDNA following the manufacturer's instructions. Finally, the target sequence was amplified. The amplification primers for CXCR4, CD70, and CD90 mRNA were displayed in Table 3. The experiment was performed with a LightCyder 480 Real‐Time PCR system (Roche). Then the relative expressions of mRNA were calculated. The results of the qRT‐PCR were analysed using the pairwise fixed reallocation randomization test.

**TABLE 3:** Sequences of the PCR amplification primers

Targets	Primer sequence(5′‐3′)
(1) CXCR4	F:TTCTACCCCAATGACTTGTG; R:ATGTAGTAAGGCAGCCAACA
(2) CD73	F:CAAATCCCACACAACCACTG; R:TGCTCACTTGGTCACAGGAC
(3) CD90	F:GACCCGTGAGACAAAGAAGC; R:GCCCTCACACTTGACCAGTT
(4) GAPDH	F:CATCTCTGCCCCCTCTGCTG; R:GCCTGCTTCACCACCTCTTG

### 2.10 Statistical analysis

IBM SPSS Statistics for Windows v22.0 (IBM Corp.) was used for data analysis. All continuous data were expressed as the mean ± standard deviation. One‐way analysis of variance and Chi‐square test were used for the statistical evaluation. The statistical significance level was set at *P* value < .05.

## 3 RESULTS

### 3.1 Characterization of microbubbles

**FIGURE 2:**
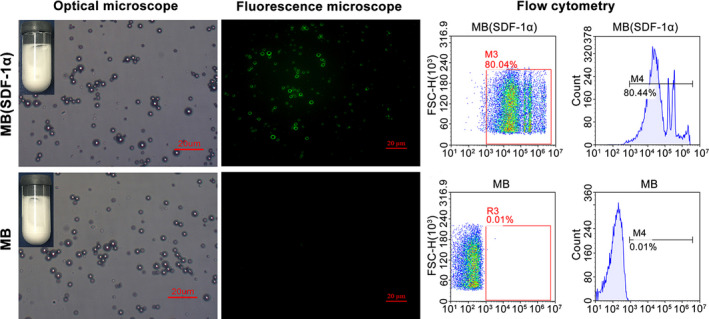
Characteristics of microbubbles. MB(SDF‐1α) and MB under the optical microscope presented uniform size and distribution. MB(SDF‐1α) under inverted fluorescence microscope exhibited obvious fluorescence while MB had no fluorescence. Flow cytometry of two kinds of microbubbles revalidated the presence of SDF‐1α on the surface of MB(SDF‐1α)

We have successfully prepared ordinary microbubbles MBs and chemotactic microbubbles MB(SDF‐1α). The two kinds of microbubbles were similar on an unaided eye and presented as milky suspensions (Figure 2). They were stable at room temperature and there was no statistical difference in the count and particle size of the microbubbles in a short period of time (Table 4).

**TABLE 4:** Beckman Coulter determination of concentration and particle size of microbubbles

Time	Concentration (×10^8^/mL)			Particle size (µm)		
	MB(SDF‐1α)	MB	*P* value	MB(SDF‐1α)	MB	*P* value
2 h	2.54 ± 0.28	2.96 ± 0.11	.138	1.624 ± 0.268	1.498 ± 0.287	.107
8 h	2.39 ± 0.62	2.62 ± 0.21	.066	1.702 ± 0.293	1.510 ± 0.216	.114
24 h	2.21 ± 0.40	2.33 ± 0.24	.071	1.722 ± 0.604	1.645 ± 0.448	.092
*P* value	.278	.312		.094	.118	

Observed under the optical microscope, both MB(SDF‐1α) and MB showed a spherical structure with uniform size and uniform distribution (Figure 2). And both microbubbles showed irregular motion. Under an inverted fluorescence microscope, the green fluorescence expressed by FITC was observed on the surface of MB(SDF‐1α), while MB showed no green fluorescence (Figure 2). Flow cytometry indicated that the carry rate of FITC fluorescence was greater than 80% (Figure 2). The SDF‐1α encapsulation efficiency was 76%, and the loading content was 15 μg/mL.

### 3.2 BMSCs migration in vitro

**FIGURE 3:**
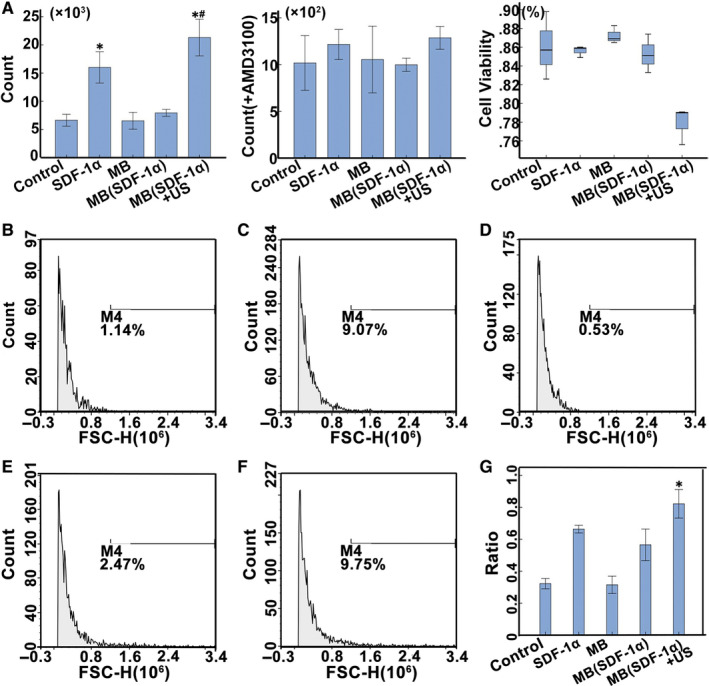
Stem cell in vitro migration assay. A, The number of cell migrations in each group in chemotaxis experiment and chemotaxis inhibition experiment. In the chemotaxis experiment, SDF‐1α and MB(SDF‐1α) groups had higher cell counts than the other groups (*), and the MB(SDF‐1α) group had the highest cell count (#). After blocking with AMD, there was no significant difference in the number of migrated cells in each group. Cell viability assessment of the number of migrated cells in each group in the chemotaxis experiment. There was no statistical difference in cell viability among the groups. B‐F (Group 1‐5), Flow cytometry was used to detect the expression of CXCR4 on the surface of BMSCs in each group. Group 2 and 5 had a relatively higher expression. G, qRT‐PCR of CXCR4 mRNA, and group MB(SDF‐1α) + US had the highest expression (*)

In the chemotaxis experiment and chemotaxis inhibition experiment, the number of cells in the lower chamber of each experimental group was detected using a fully automatic cell counter. We found that the number of migrated cells in SDF‐1α group and MB(SDF‐1α) + US group was higher than that in the other three groups, and MB(SDF‐1α) + US group was the highest in chemotaxis experiment (*P* < .05), while in the chemotaxis inhibition experiment, the number of migrated cells in each group was significantly lower than that in the absence of antagonists AMD3100. And there was no significant difference in the number of migrated cells in each group (*P* > .05; Figure 3A). In chemotaxis experiment, cell viability of each group showed that MB(SDF‐1α) + US group was slightly lower than that of other groups, but there was no statistically significant difference (Figure 3B‐F). The expression of CXCR4 was evaluated by flow cytometry and qRT‐PCR. Flow cytometry indicated that group SDF‐1α and MB(SDF‐1α) + US showed relatively higher expression of CXCR4, while qRT‐PCR determined that group MB(SDF‐1α) + US had the highest relative expression (Figure 3G).

### 3.3 Evaluation of cartilage lesions in vivo

**FIGURE 4:**
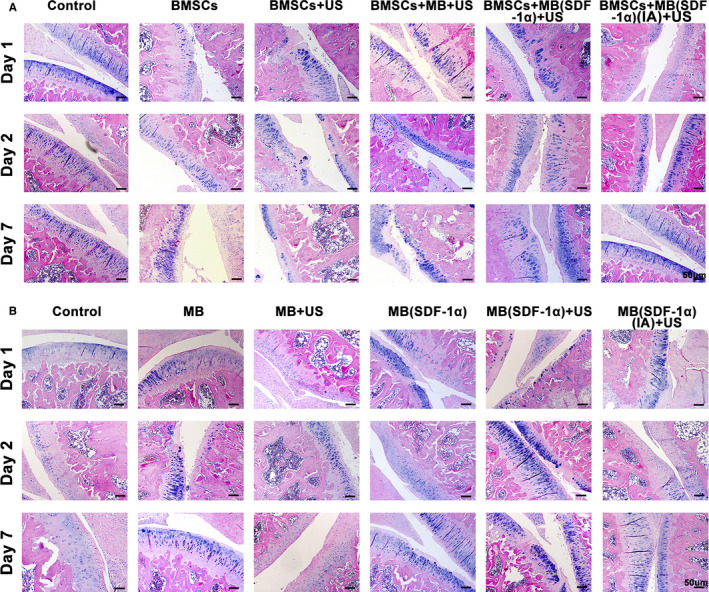
A, HE staining of each group in verification of exogenous stem cell homing (Exogenous BMSCs group). B, HE staining of each group in verification of possible endogenous stem cell homing (Non‐Endogenous BMSCs group)

A certain degree of cartilage defects was observed in HE staining of each group in the experiment of exogenous stem cell group (Figure 4A) and non‐exogenous stem cell group (Figure 4B), respectively. Synovial hyperplasia and inflammatory cell infiltration could be found in joint cavities of some groups based on HE staining. On one hand, the successful establishment of the OA model was verified. On the other hand, it was also demonstrated that after different experimental treatments, the degree of inflammation of the joints between the groups did not change significantly.

**FIGURE 5:**
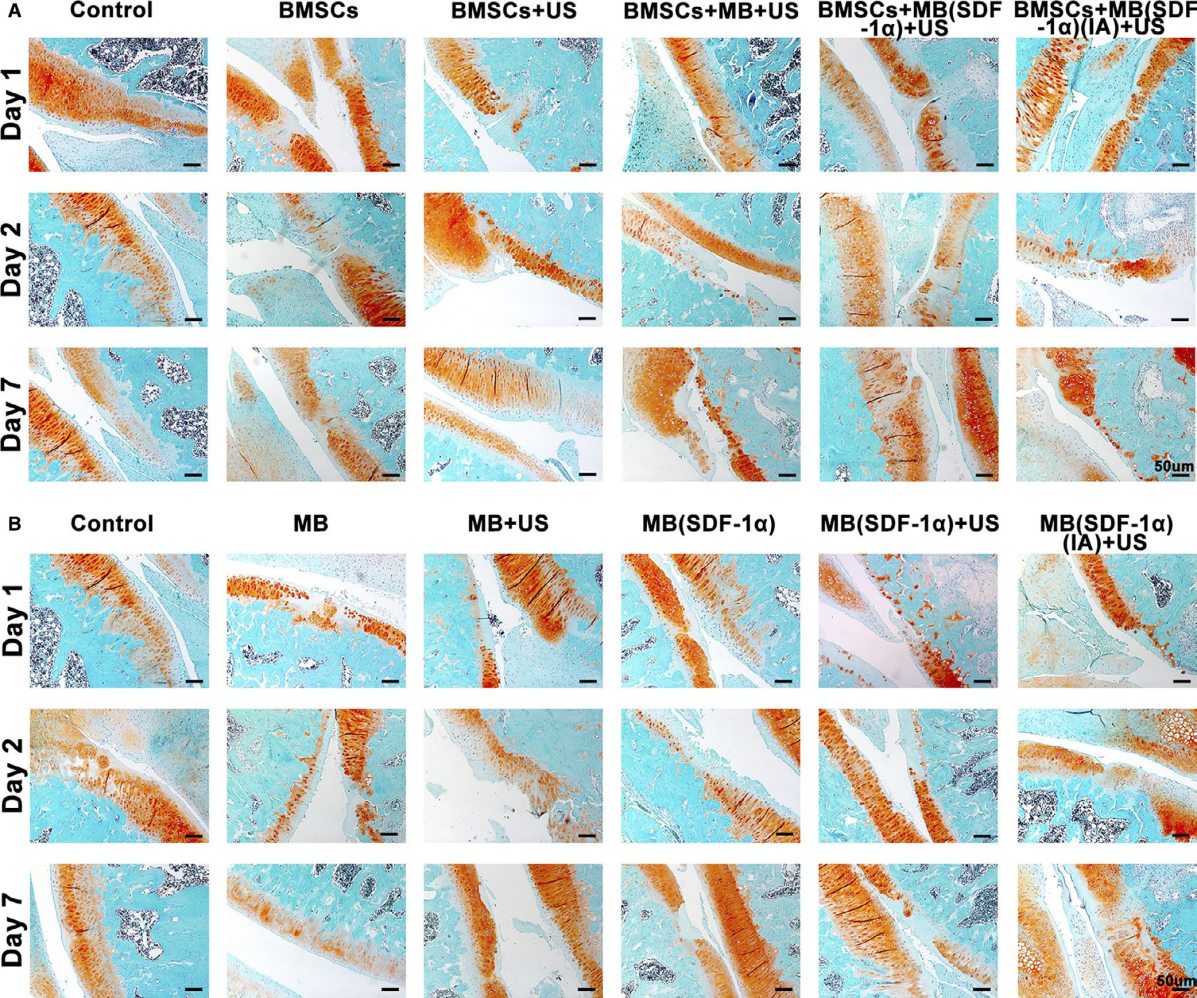
A, Reddish O‐fixed green staining of each group in verification of exogenous stem cell homing (Exogenous BMSCs group). B, Reddish O‐fixed green staining of each group in verification of possible endogenous stem cell homing (Non‐Endogenous BMSCs group)

Safranin O‐Fast green staining of experiments of verification of exogenous stem cell group (Figure 5A) and non‐exogenous stem cell group (Figure 5B) were as follows. Markin's scoring derived from Safranin O‐Fast green staining in different groups had no significant difference (*P* > .05) by scoring the cartilage structure damage, number of chondrocytes, degree of stroma staining and integrity of the tidal line.

### 3.4 Detection of BMSCs surface markers in vivo

#### 3.4.1 IHC

**FIGURE 6:**
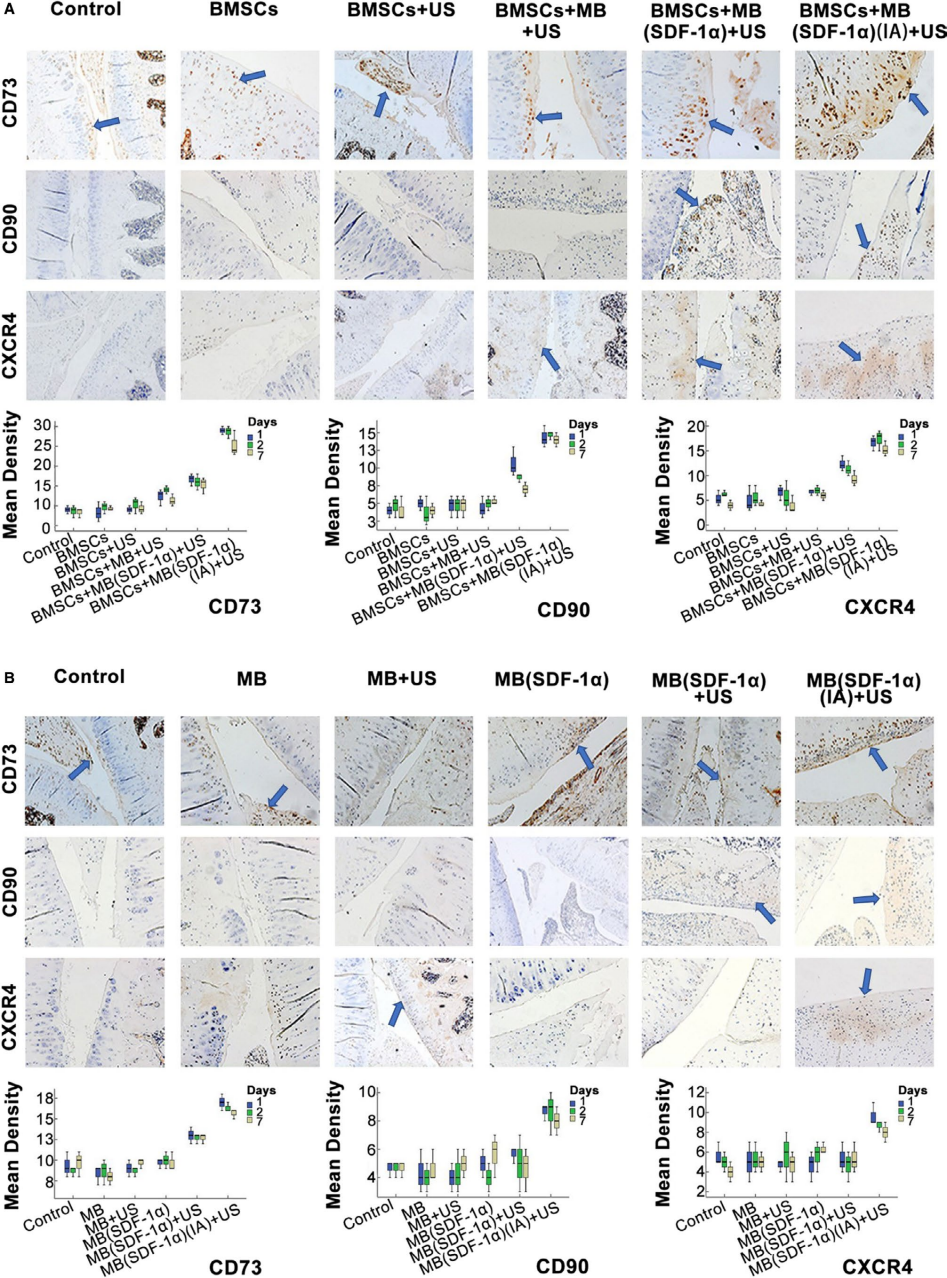
A, IHC staining of CXCR4, CD73, CD90 in exogenous BMSCs groups and their quantitative analysis. B, IHC staining of CXCR4, CD73, CD90 in Non‐Endogenous BMSCs groups and their quantitative analysis. The blue arrows indicated the positive areas

In the experiment of verification of exogenous stem cell homing, the IHC results of CD73, CD90 and CXCR4 showed that the staining intensity of BMSCs + MB(SDF‐1α) (I) + US group was higher than that of intravenous injection, and the staining intensity of BMSCs + MB(SDF‐1α) + US group was higher than the other intravenous injection groups (*P* < .05; Figure 6A).

While in the experiment of verification of endogenous stem cell homing, the IHC staining of CD73, CD90 and CXCR4 in each group was lower than that in the local injection group (*P* < .05). Except for the blank control group, the staining intensity of each group in verification of exogenous stem cell homing was higher than that in the endogenous stem cell groups (Figure 6B).

#### 3.4.2 qRT‐PCR

**FIGURE 7:**
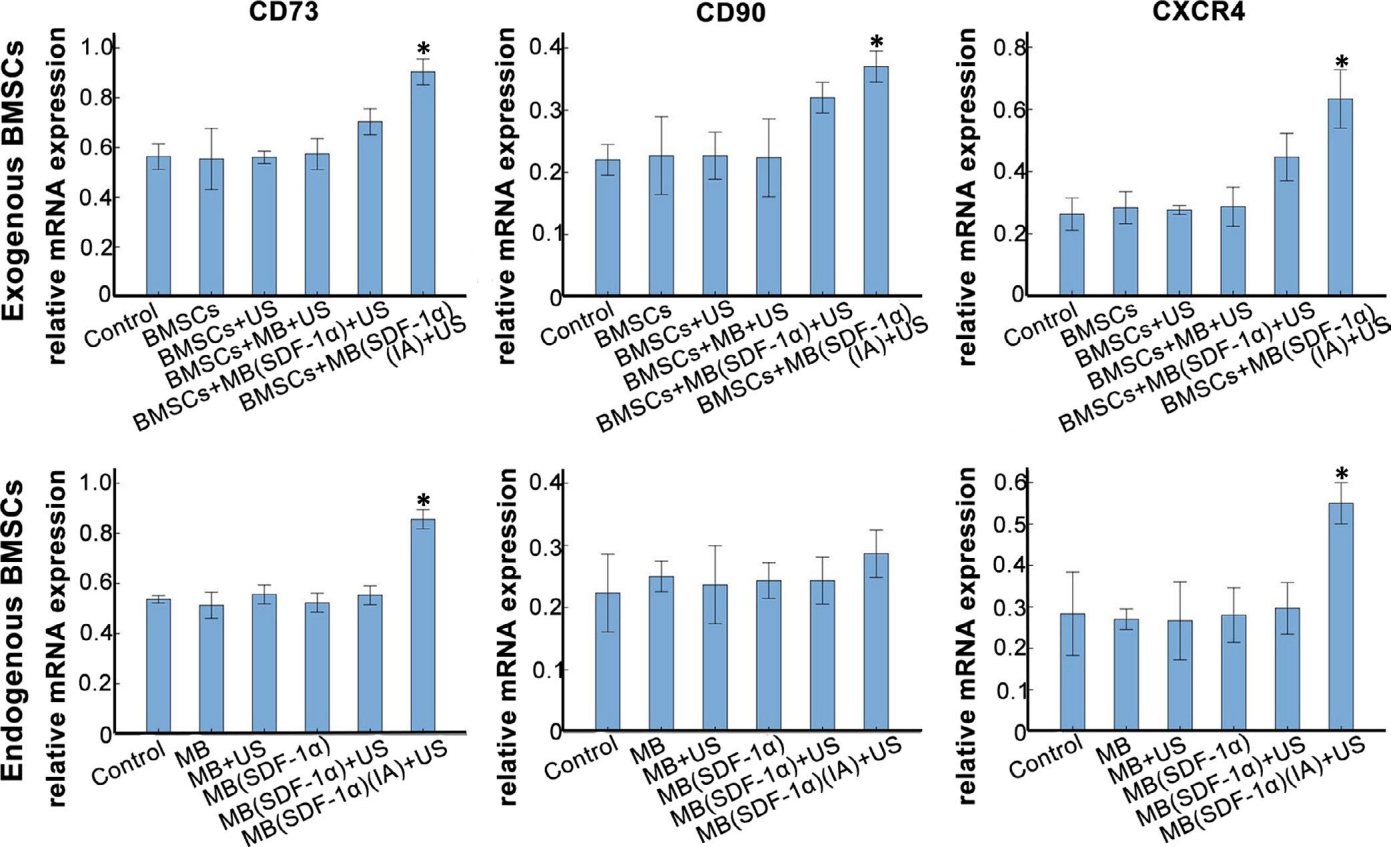
qRT‐PCR analysis of the expression levels of CXCR4, CD73 and CD90 mRNA.* represents a statistically significant difference

The expression levels of CXCR4, CD73 and CD90 mRNA in BMSCs + MB(SDF‐1α) (IA) + US group were higher than those in the other groups in exogenous stem cell homing experiment (*P* < .05). And CXCR4, CD73 mRNA expression level in MB(SDF‐1α) (IA) + US group without exogenous BMSCs was higher than the other groups (*P* < .05; Figure 7), which indicated the possibility of endogenous BMSCs homing.

## 4 DISCUSSION

Current delivery strategies of MSCs mainly include local injection and systemic injection. As OA is a systemic disease, multiple joints are often involved instead of a single joint. Local injections are limited to treat multiple joints simultaneously. In addition, systemic injection of stem cells is beneficial for verifying the homing of stem cells. Therefore, this study attempted to use systematic administration of stem cells for research.

In this research, we have successfully developed chemotactic microbubbles containing SDF‐1α by covalent conjugation. The MB(SDF‐1α) has good stability and has no significant change in particle size and count in a short time with homogeneous distribution. This may be caused by the mixed phospholipids that used as shell materials for chemotactic lipid microbubbles, which has better stability than a single phospholipid shell. The PEG‐loaded phospholipids can protect the microbubbles in the blood circulation system, thereby forming a protective layer of hydration film on the surface of the microbubbles, further promoting the stability of the microbubbles. It's still better to prepare them before use in order to achieve the best performance of the microbubbles. The prepared MB(SDF‐1α) also has a high unloading content and encapsulation efficiency of SDF‐1α. In addition, the MB(SDF‐1α) also has a satisfactory FITC fluorescence unloading rate as SDF‐1α was labelled with FITC prior to use. The result is comparable to the study of Wu and his group.^13^

In the chemotaxis experiment in vitro, the MB(SDF‐1α) solution significantly promoted the migration of BMSCs, and it has better chemotaxis than the equivalent amount of SDF‐1α alone. While in the chemotaxis inhibition experiment, the migration efficiency was restrained after the block of the SDF‐1/CXCR4 axis by AMD3100, which is a highly specific receptor antagonist of CXCR4. In the bioactivity assay in vitro, the BMSCs in each group had similar bioactivity and did not show a large number of cell deaths due to ultrasound irradiation. This indicated that the ultrasonic irradiation conditions of duty ratio 10%, intensity 1 W/cm^2^, time 30 seconds were relatively safe for BMSCs.

The CXCR4 receptor expressed on the cell membrane of BMSCs is usually less, and it is further decreased as the culture time in vitro is prolonged and the number of passages is increased. The expression of CXCR4 directly affects the binding to SDF‐1α, which in turn affects the migration of stem cells. This is the reason for evaluating the expression of CXCR4 on the surface of BMSCs and selecting P_3_ BMSCs for experiments. Both flow cytometry and qRT‐PCR revealed that the expression of CXCR4 in MB(SDF‐1α) + US group was relatively higher than the other groups.

In the experiments verifying exogenous BMSCs homing (exogenous BMSCs injected) and possible endogenous BMSCs (non‐exogenous BMSCs injected) homing in vivo, a certain degree of cartilage defects appeared in each group, which was consistent with the model of OA. There were no significant differences in Markin's scores and degree of cartilage damage in each group.

The staining intensities of CXCR4, CD73, and CD90 were detected by IHC staining of articular cartilage in the lesion area. And qRT‐PCR was used to detect the relative expression of each mRNA. When exogenous BMSCs were administrated, the expression of stem cell positive markers and chemokine receptors in BMSCs + MB(SDF‐1α) (IA) + US group were significantly higher than the other groups. It was confirmed that the local stem cell content of this group was higher than the other groups, and the stem cells had the highest homing efficiency. At the same time, the IHC staining intensity of BMSCs + MB(SDF‐1α)(iv) + US group was higher than that of the other intravenous injection groups, which also showed that in all intravenous injection groups, the combination of ultrasound and chemotactic microbubbles promoted stem cell homing with best efficiency. In the study of possible endogenous stem cell homing, MB(SDF‐1α) (IA) + US group also caused local increase in stem cell surface markers and chemokine receptor levels, which also verified that ultrasound combined with chemotactic microbubbles could induce endogenous stem cells to homing to target tissues in a certain degree. Marrow‐stimulating procedures are directed at mobilizing endogenous MSC to the site of cartilage injury, capitalizing on the intrinsic healing abilities of the body.^29^ This part of the stem cells may come from the synovial membrane, bone marrow, blood, etc However, the role of endogenous stem cell homing is limited, and the intensity of IHC staining and mRNA expression level is not as good as those of exogenously administrated stem cells. We have been suggested that in the experimental group of exogenously injected stem cells, ultrasound combined with chemotactic microbubbles MB(SDF‐1α) may also promote the homing of some endogenous stem cells. Similar conclusion was made by another studies suggesting that trafficking of native MSC to injured tissue and their subsequent participation in the regenerative process is a natural healing response, which can potentially be imitated or augmented by enhancing the endogenous MSC pool with exogenously administered MSC.^29^ In this way, after injecting exogenous MSC, both of exogenous and endogenous MSC can play a synergistic role in cartilage repair.

The common administration ways of exogenous MSC include local injection (intra‐articular injection for joints) and intravenous injection. Between them, local injection of MSC is the simplest and most direct way for the supply of MSC. Because of the lack of vascularity in cartilage, there is limited researches focused on that MSC (from the circulation) are capable of homing in response to cartilage injury by intravenous injection except for Pereira and his group.^29^ In this study aimed at investigating whether precursor mesenchymal cells from the marrow that are expanded in culture can serve as long‐lasting precursors of bone and other connective tissues after intravenous infusion into irradiated mice, Pereira et al^30^ reported that progeny of the donor cells exogenously delivered were present in cartilage and accounted for 2.5% of the isolated chondrocytes from xiphoid and articular cartilage, which demonstrates the possibility of injecting stem cells intravenously. Further studies are still needed for the intravenous administration of MSC.

Although Saito et al^31^ have demonstrated the ability of MSCs to home for the first time since 2002, the exact mechanism of MSCs therapy is still controversial. Currently, the main possible mechanisms are as follows. First, the SDF‐1/CXCR4 axis is the most important biological axis for promoting stem cell homing.^15^, ^16^ On one hand, it can promote the migration of BMSCs to the injured part after transplantation. On the other hand, when the tissue is damaged, it will regulate the expression of SDF‐1α, and the local SDF‐1α concentration will increase. Then BMSCs containing CXCR4 on the cell surface will reach the injury site along the concentration gradient of SDF‐1α and participate in the tissue repair process. Second, MSCs have chemotaxis similar to immune cells and chemotaxis to damaged or inflammatory regions; and third, MSCs are similar to the vascular endothelial cells, and they can be implanted in the basement membrane of systemic microvasculature or capillaries to enter the target organ.^32^

The combination of ultrasound and microbubbles can cause sonoporation under proper conditions. With the effect of sonoporation, transient and non‐lethal porosity will occur on biological membranes, which will increase the permeability of capillaries.^33^, ^34^ In addition, Meijering et al^35^ proposed that the microbubbles rupture caused the generation of hydrogen peroxide with ultrasound irradiation. And it also resulted in Ca^2+^ influx and Ca^2+^‐dependent K channel opening in the adjacent cell membrane, thereby causing local membrane potential hyperpolarization. Endocytosis and pinocytosis can help the microbubble get into the cell under ultrasound irradiation.

This preliminary research still has several limitations. We have no traces of exogenous stem cells in this study. In addition, there is no further study on the long‐term survival and differentiation of exogenous stem cells. This will be covered in our future research.

## 5 CONCLUSION

We successfully prepared a chemotactic microbubble MB(SDF‐1α) which combining microbubble with SDF‐1α by covalent conjugation. It has good stability and high unloading content and encapsulation efficiency of SDF‐1α. Ultrasound combined with MB(SDF‐1α) can promote the migration of BMSCs in vitro and also improve the homing of BMSCs in vivo. When intravenously injected with exogenous stem cells, ultrasound combined with MB(SDF‐1α) in intra‐articular injection promoted the best homing of stem cells to the cartilage defect area. While if exogenous BMSCs were not given, ultrasound combined with MB(SDF‐1α) intra‐articular injection also promoted the homing of endogenous BMSCs to a certain extent, however, the homing efficiency was inferior to the administration of exogenous BMSCs. Ultrasound combined with chemotactic microbubble MB(SDF‐1α) is an effective way to promote stem cell homing in the rat OA model, which is of great significance for cartilage regeneration.

## CONFLICT OF INTEREST

The authors confirm that there are no conflicts of interest.

## AUTHOR CONTRIBUTIONS

**Xi Xiang:** Data curation (lead); Writing‐original draft (lead). **Hui Liu:** Data curation (supporting); Methodology (supporting). **Liyun Wang:** Methodology (supporting). **Bihui Zhu:** Methodology (supporting). **Lang Ma:** Supervision (equal); Writing‐review & editing (equal). **Fangxue Du:** Methodology (supporting). **Ling Li:** Data curation (supporting). **Li Qiu:** Conceptualization (lead); Funding acquisition (lead); Resources (lead); Supervision (equal); Writing‐review & editing (equal).

## Data Availability

All data were included in the manuscript.
