# Correction to Ultrasound combined with SDF‐1α chemotactic microbubbles promotes stem cell homing in an osteoarthritis model

**DOI:** 10.1111/jcmm.17776

**Published:** 2023-07-14

In this article by Xi Xiang et al.,^1^ there were errors in the images of Figure 4A and Figure 5.

In Figure 4A, the HE staining of Day 1 in control group and Day 7 in BMSCs + MB(SDF‐1α)(IA) + US group were exactly the same. The corrected Figure 4A is below.

**Figure:**
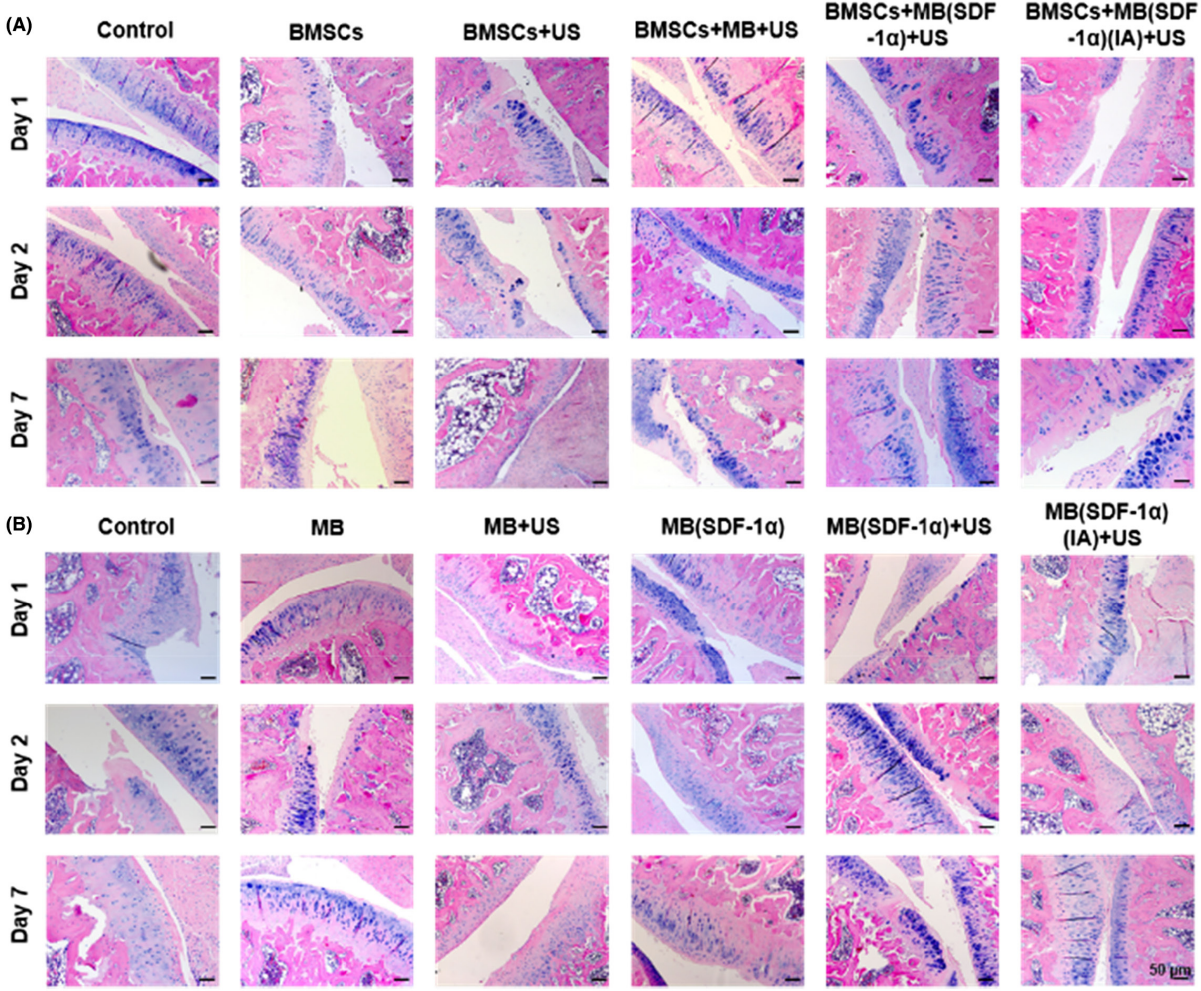
Figure 4

In Figure 5, the reddish O‐fast green staining of Day 2 in control group and Day 1 in control group were exactly the same. Day 2 control group was mistakenly placed in Day 7 control group. The corrected Figure 5 is below.

**Figure:**
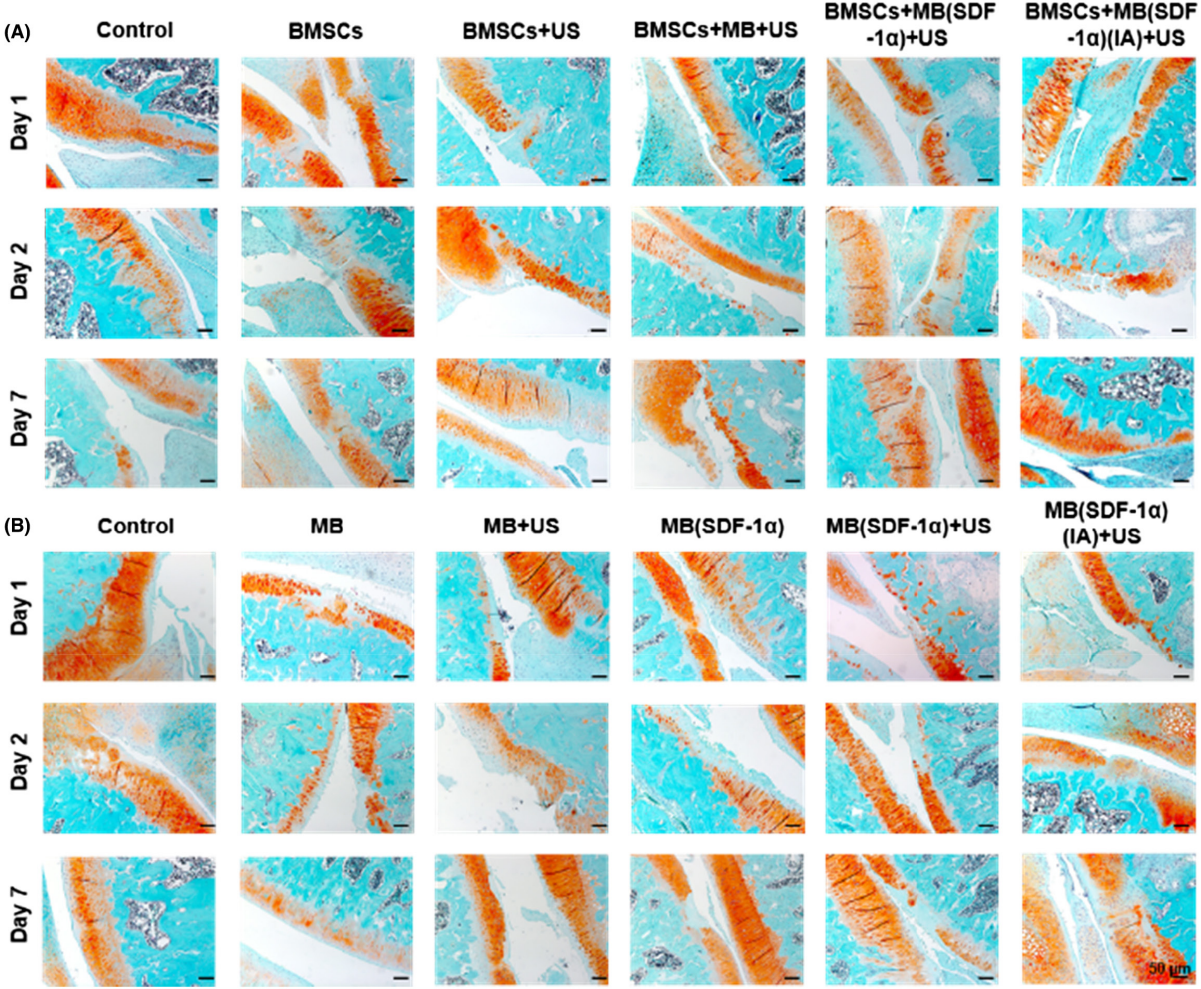
Figure 5

The authors confirmed that all results and conclusions of this article remain unchanged.

We apologise for these errors.
